# Ureteral wall thickness at the ureterovesical junction as a significant factor in predicting medical expulsive therapy of ureterovesical junction stones

**DOI:** 10.1186/s12894-025-01890-9

**Published:** 2025-08-23

**Authors:** Tiancan Yang, Jian Ji, Yafei Wang, Xiaowen Gao, Lingmin Lei, Lvyang Chen, Shicheng Fan, Zhida Wu, Wei Pu, Yunbo Shang

**Affiliations:** 1 Department of Urology, the Second Affiliated Hospital of Dali University (the Third People’s Hospital of Yunnan Province), Kunming, 650011 China; 2 Department of Urology, the Third People’s Hospital of Yunnan Province, No. 292 Beijing Road, Guandu District, Kunming, 650011 China; 3 Department of Surgery, the Fifth People ’s Hospital of Yueqing City, No. 66, Xiazhaiyang Road, Dajing Town, Yueqing City, Wenzhou, 325615 China

**Keywords:** Ureterovesical junction stones, Ureteral wall thickness, Medical expulsive therapy

## Abstract

**Background:**

To evaluate ureteral wall thickness (UWT) at the ureterovesical junction (UVJ) measured by ultrasound for predicting spontaneous passage (SP) of uncomplicated UVJ stones.

**Patients and methods:**

We retrospectively reviewed 170 patients ≥ 18 years old, and size ≤ 10 mm of single UVJ stone, who were examined and treated in the Third People’s Hospital of Yunnan Province from January 2020 to January 2024. The analysis included the size of the stones, the maximum UWT at the stone site measured by ultrasound, the degree of hydronephrosis, and the time of stone removal.According to the different results after four weeks of medical expulsive therapy (MET), the patients were separated into two categories: Stone-passing group (SPG) and non-stone-passing group (NSPG). Univariate and multivariate logistic regression analysis were utilised to evaluate the clinical predictors of MET.The receiver operating characteristic (ROC) curve was employed to evaluate the accuracy of the UWT at the UVJ in predicting successful MET.

**Results:**

The SPG comprised 112 cases (65.9%), while the NSPG consisted of 58 cases (34.2%). Univariate analysis, employing both the chi-square test and the Mann-Whitney U test, revealed that gender, age, stone side and degree of hydronephrosis were not statistically significant. However, stone size and UWT were found to be influencing factors in regard to stone removal. Binary logistic regression analysis demonstrated that UWT and size were independent influencing factor of MET. The ROC analysis indicated that 3.705 mm was the ideal threshold for UWT, with sensitivity and specificity levels of 72.4% and 68.7%, with an area under the ROC curve (AUC) of 0.737.

**Conclusions:**

The UWT at the UVJ has a high predictive value for the MET of stones at this position, thus avoiding the adverse consequences of delayed stone discharge caused by unnecessary surgical operation and MET.

## Background

Ureteral stones are a common urological disease, and their diagnosis and treatment have always been a focus in clinical practice. Accurate assessment of UWT is crucial for predicting the success rate of stone passage and optimizing treatment plans. However, traditional non-contrast spiral CT (NCCT), although highly sensitive and specific for stone detection, has limitations in measuring the UWT at the UVJ. The complex anatomical structure of this area, along with interference from the bladder, makes it difficult and inaccurate for NCCT to measure the UWT. In this study, we used ultrasound to measure the wall thickness at the UVJ, successfully solving the problem of the difficulty in measuring with NCCT. For stones located at the UVJ, there are several treatment options, primarily divided into conservative treatment and surgical management. These approaches lead to varying levels of patient benefit and associated risks. The goal of MET is to relieve pain, facilitate stone passage, and prevent factors that impede stone expulsion, such as ureteral edema, spasms, and infections [1]. Non-steroidal anti-inflammatory drugs and α-blockers are commonly used in the conservative treatment of ureteral stones [2]. The physiological effect of α-blockers on the smooth muscle layer of the ureter is significant, as α-adrenergic receptors are more densely distributed in the distal third of the ureter [3].

As a continuation of the ureter in the bladder, UVJ represents the narrowest physiological segment of the ureter [4–6]. Most studies have focused on the impact of UWT and stone size on surgical or medical treatments for ureteral stones. However, there is a relative lack of studies specifically addressing the anatomical segment of the UVJ. Therefore, it is crucial to investigate the factors influencing MET for stones located at the UVJ.

## Materials and methods

We analyzed the data of 170 patients diagnosed with single, ≤ 10 mm ureterovesical junction stones who received MET (tamsulosin) treatment between January 2020 and January 2024. Inclusion criteria: clinically and radiologically diagnosed UVJ stones, single stones of ≤ 10 mm, conservative treatment with oral tamsulosin (0.2 mg once daily) for 4 weeks, and no prior treatments. Exclusion criteria: prior treatment, history of urinary stone surgery, renal insufficiency, congenital urinary anomalies or malignancy.

Before starting MET, patients were thoroughly informed about the treatment steps and potential complications, such as delayed stone passage leading to severe hydronephrosis, infection, and pain. Patients who agreed to participate signed informed consent and were followed up regularly to monitor stone SP. During the treatment process, in addition to the routine use of tamsulosin, we also administered nonsteroidal anti-inflammatory drugs (ibuprofen) based on the specific conditions of the patients.

**Fig. 1:**
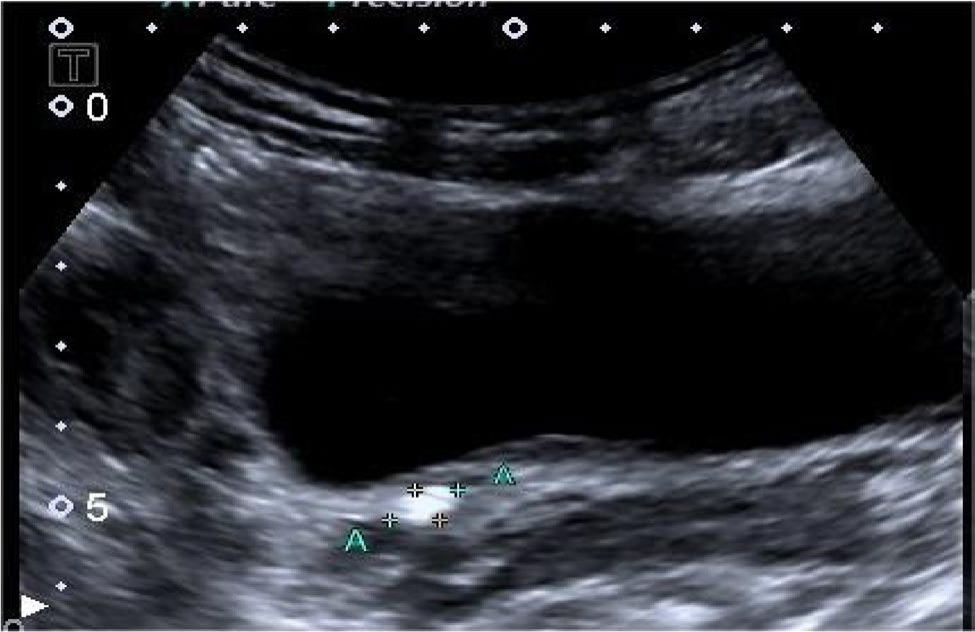
Ultrasound image of ureterovesical junction calculi

**Fig. 2:**
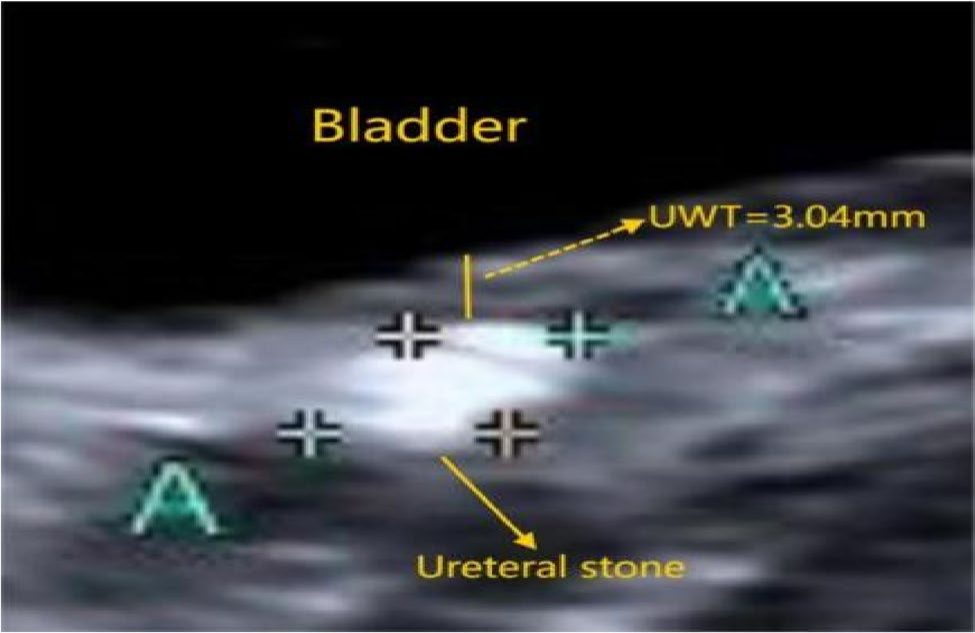
The stone is enlarged in Fig.a, and the gap between Bladder and Ureteral stone is UWT

Data collected included patient age, sex, stone laterality, the use of anti-inflammatory drugs. hydronephrosis grade (Society for Fetal Urology grading system [7]), stone size, and the UWT at the stone site. All radiological parameters were derived from ultrasound examinations. Due to the unique anatomical location of the UVJ, we measured the UWT by ultrasound, with the anterior wall thickness substituted for the measurement of the entire ureteral segment due to difficulty visualizing the posterior wall (Figs. 1 and 2). The ultrasound images were obtained by qualified technicians, and all measurements were performed using ImageJ software with a scale from the collected ultrasound images.

### Statistical analysis

The data were analyzed using SPSS version 26.0. The Chi-square test was used for categorical variables, while the Mann-Whitney U test was applied for continuous variables. Factors with significant differences in univariate analysis were subjected to binary logistic regression. ROC curve analysis was performed to assess the accuracy of UWT in predicting successful stone expulsion with MET.

## Results

A total of 170 patients with UVJ stones were included in the study, with a stone expulsion rate of 65.9%. Based on the outcomes of MET, the patients were divided into two groups: SPG (112 patients) and the NSPG (58 patients). The two groups did not show significant differences in gender, age, stone laterality, use of Ibuprofen (yes/no), or degree of hydronephrosis (*p* > 0.05) (Table 1).

Univariate analysis revealed significant differences between the SPG and NSPG in terms of stone size and UWT (Table 1). Following the univariate analysis, binary logistic regression was conducted, which indicated that stone size (*p* = 0.004) and UWT (*p* < 0.001) are independent factors influencing stone SP (Table 2).

**Fig. 3:**
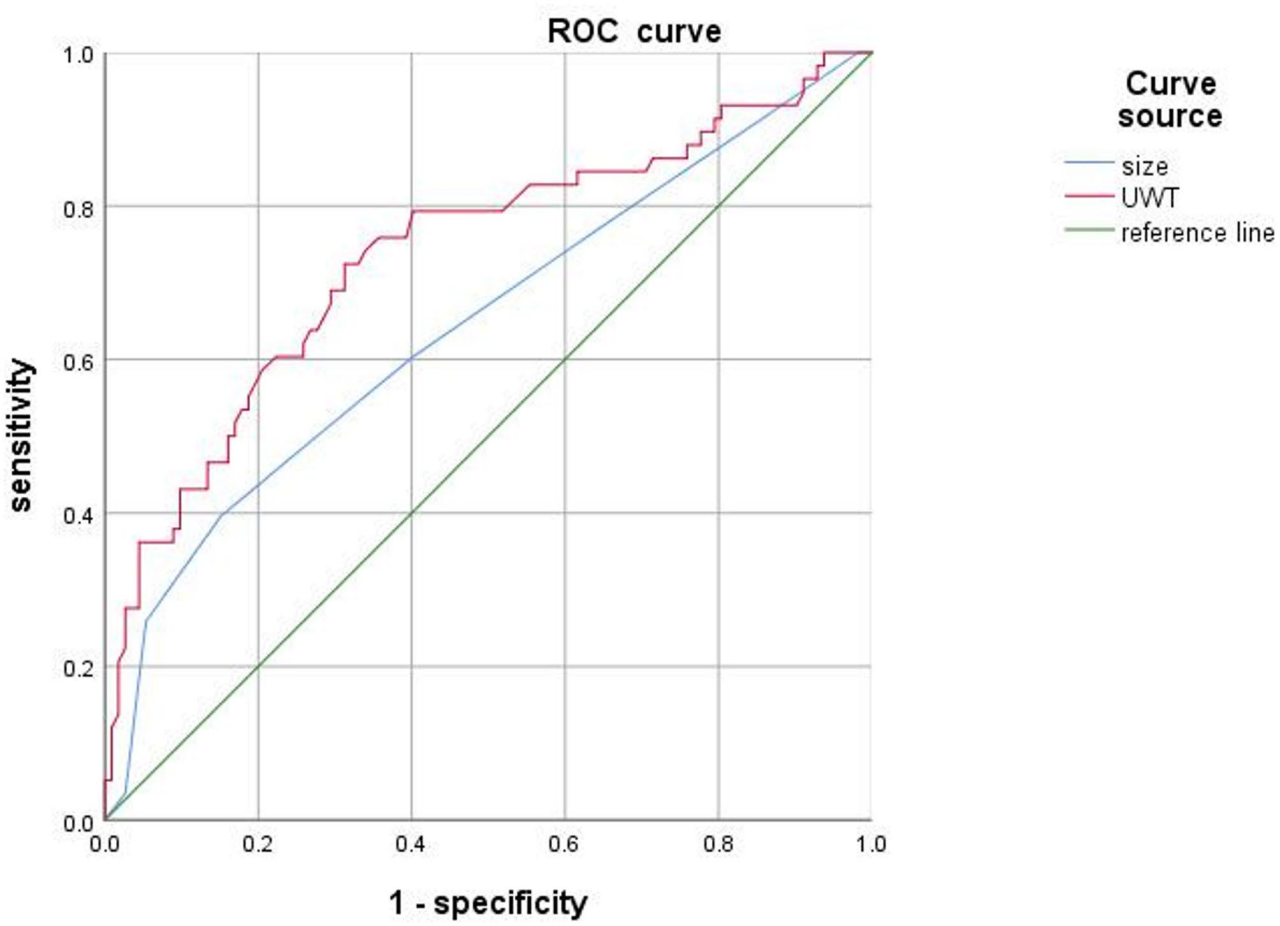
ROC curve of logistic model prediction

ROC curve analysis demonstrated that UWT could be used as a predictive factor for the success of MET. The AUC for UWT was 0.737 (Fig. 3), with 3.705 mm was a ideal threshold for UWT. That means that when the UWT of the stone is ≥ 3.705 mm, the stone may spontaneously fail, which demonstrated a sensitivity of 72.4% and specificity of 68.7%. And the AUC for stone size is only 64.5%.

**Table 1:** Univariate analysis of stone-related variables in the two groups

	Total	Stone-passing group	non-stone-passing group	*P* value
Gender				0.375
Male	131	84	47	
Female	39	28	11	
Age, years		40.(29.25,50.75)	45(32,59.75)	0.069
Side				0.230
Left	90	63	27	
Right	80	49	31	
Oral NSAIDS (Ibuprofen) taken during MET				0.343
Yes	140	90	50	
No	30	22	8	
Stone size (mm)		6(5,7)	7(6,9)	0.002
UWT (mm)		3.435(3.0525,0.8500)	3.97(3.62, 4.8225)	<0.001
Hydronephrosis				0.436
Grade 0	74	51	23	
Grade 1	91	59	32	
Grade 2	4	2	2	
Grade 3	1	0	1	
Grade 4	0	0	0	

**Table 2:** Multivariate logistic regression analysis

Variable	OR(95%CI)	*P* value
Stone size (mm)	1.407(1.116–1.774)	0.004
UWT (mm)	3.214(1.936–5.333)	<0.001

## Discussion

Ureteral stones account for approximately 20% of all urinary tract stones [8], with distal ureteral stones representing 70% of all ureteral stones [9]. Previous studies have shown that there are three physiological strictures in the upper urinary tract. However, recent advances in medical imaging technologies and their application in clinical anatomy have challenged this long-standing theory. Minobu Kamo et al. [10] demonstrated that the most common sites of obstruction are limited to two locations: the renal pelvis-ureter junction and the UVJ. As the most narrow anatomical point in the ureter, UVJ is also the main site of stone retention [10]. The passage of ureteral stones through the UVJ leads to ureteral edema, hypertrophy, interstitial fibrosis, and a cascade of pathological alterations [11]. These changes contribute to the development of clinical manifestations such as pain, fever, and urinary tract obstruction, which not only complicate treatment but also significantly impair the patient’s quality of life.

Surgical treatment of distal ureteral stones primarily includes extracorporeal shock wave lithotripsy (ESWL) and ureteroscopic lithotripsy (URSL) [12–14]. Despite significant advancements in surgical techniques, complications such as infection, ureteral injury, and anesthesia-related risks continue to pose substantial challenges. Moreover, both ESWL and URSL are expensive treatment modalities, which not only result in physical harm but also impose a significant economic burden on patients [15].

In the past few years, the efficacy of MET for distal ureteric stone is controversial. However, According to guidelines and studies, patients patients with uncomplicated ureteral stones ≥ 5 mm and ≤ 10 mm in size should be offered conservative treatment including observation or MET [16–18]. The α-adrenergic receptors in the distal third of the ureter (including the UVJ) are more densely distributed than in the remaining of the ureter [4–6]. α-blockers have been shown to reduce ureteral smooth muscle tone, thus facilitating stone passage and expulsion [19]. Compared to surgical interventions, MET reduces healthcare costs and associated risks. UWT has been widely used as a preoperative predictor of stone passage in ureteral stones [11, 20, 21]. Additionally, some studies have utilized UWT to predict the stone expulsion rate following MET [9, 22]. However, to date, no literature has reported the predictive value of UWT at the UVJ for MET.

In the present study, there was no significant difference between gender, age, stone laterality, the use of anti-inflammatory drugs and hydronephrosis severity with the outcomes of MET (*p* > 0.05). Conversely, Yoshida et al. [23] reported a significant association between the degree of hydronephrosis and stone passage in their univariate analysis. However, in their multivariate analysis, hydronephrosis severity was not identified as an independent factor influencing stone expulsion. Similarly, Mohamed Samir et al. [9] found a significant correlation between the degree of hydronephrosis and stone expulsion in both univariate and multivariate analyses. The discrepancy between our findings and those of Yoshida and Samir may be attributed to the different sample size and most cases presenting mild hydronephrosisin our study.

In our study, the results of the multifactor analysis indicate that both stone size and UWT are independent influencing factors of MET. Stone size directly impacts the resistance to expulsion, with larger stones exerting mechanical compression on the ureteral wall, leading to ureteral injury and inflammation. This inflammatory response further induces edema or hyperplasia of the ureteral wall, narrowing the ureteral lumen and consequently hindering the mobility of the stone, thereby increasing the risk of stone impaction and ultimately reducing the success rate of MET. Although stone size demonstrated some predictive capacity, the AUC for stone size was only 64.5%, this suggests that while there is a correlation between stone size and the success of stone passage, stone size alone is not a robust predictor of stone passage outcomes. This highlights the limited predictive power of stone size for MET success, while also acknowledging that it retains some reference value. The UWT in the SPG was significantly smaller than that in the NSPG. Logistic analysis demonstrated a significant correlation between UWT at the UVJ and the success rate of MET, with an AUC of 0.737. The cut-off value for predicting MET failure was identified as UWT ≥ 3.705 mm. Mohamed Samir et al. [9] reported a UWT cut-off value of 3.75 mm for the distal ureter, while Yoshida et al. [23] found a UWT cut-off of 2.71 mm for predicting stone passage at 4 weeks.The differences in UWT may be attributed to the anatomical differences between the UVJ and the rest of the ureter. The UVJ is not only encased by the fibromuscular Waldeyer’s sheath but also lacks the circular muscle layer that is present in the remainder of the ureter [24]. Another possible reason is the variations in measurement methods. While our study utilized ultrasound technology, Mohamed Samir et al. [9] employed NCCT with a (7×) magnification and soft-tissue window parameters to measure UWT.

The standardized method for assessing the UWT was the use of NCCT with a(7×)magnification and soft-tissue window parameters [9, 11]. However, it should be noted that NCCT cannot accurately calculate UWT at the UVJ [9]. In the current study, we aimed to measure the UWT at the ureterovesical junction, where the ureteral orifice is located within the bladder.The posterior wall of the ureter was obscured by the strong echogenic shadow of the stones; Hence, we used the thickness of the anterior wall of the ureter to represent the UWT at the UVJ (Figs. 1 and 2). In this study, all ultrasound images were acquired by experienced ultrasound specialist. The UWT at the UVJ was measured using ImageJ software with the scale provided in the collected ultrasound images. Each image was measured three times, and the average value of these measurements was used as the final UWT value.

The primary limitation of this study is its retrospective, single-center design, which did not prospectively include parameters such as patients’ history of lower urinary tract symptoms (LUTS) and the specific use of anti-inflammatory medications. Currently, there is a lack of literature on methods for measuring the UWT at the UVJ. This study represents the first attempt to use ultrasound to address the challenge of measuring tissue thickness at the UVJ. However, we were unable to compare the thickness at the same location after stone expulsion or at the contralateral site. We hope that future research will employ a prospective design with more systematic data collection, including detailed medication records and follow-up data, and seek more effective measurement methods. Despite these limitations, we believe that our study’s data will make a meaningful contribution to ongoing research on MET for UVJ stones.

## Conclusion

The UWT at UVJ may have some predictive value for stone MET, thus avoiding the adverse consequences such as delayed stone clearance caused by MET and unnecessary surgery.

## Data Availability

The data examined in this study is available from the corresponding authors upon reasonable request.

## Abbreviations

UWT: Ureteral wall thickness
UVJ: Ureterovesical junction
SP: Spontaneous passage
MET: Medical expulsive therapy
SPG: Stone-passing group
NSPG: Non-stone-passing group
ROC: Receiver operating characteristic
ESWL: Extracorporeal shock wave lithotripsy
URSL: Ureteroscopic lithotripsy
NCCT: Non-contrast spiral CT
LUTS: Lower urinary tract symptoms
